# Dual Effect of *PER2* C111G Polymorphism on Cognitive Functions across Progression from Subjective Cognitive Decline to Mild Cognitive Impairment

**DOI:** 10.3390/diagnostics11040718

**Published:** 2021-04-18

**Authors:** Salvatore Mazzeo, Valentina Bessi, Silvia Bagnoli, Giulia Giacomucci, Juri Balestrini, Sonia Padiglioni, Giulia Tomaiuolo, Assunta Ingannato, Camilla Ferrari, Laura Bracco, Sandro Sorbi, Benedetta Nacmias

**Affiliations:** 1 Department of Neuroscience, Psychology, Drug Research and Child Health, University of Florence, 50134 Florence, Italy; salvatore.mazzeo@unifi.it (S.M.); silvia.bagnoli@unifi.it (S.B.); giuliagiacomucci.md@gmail.com (G.G.); juri.balestrini@gmail.com (J.B.); tomagiuu@gmail.com (G.T.); assunta.ingannato@unifi.it (A.I.); camilla.ferrari@unifi.it (C.F.); bracco@unifi.it (L.B.); sandro.sorbi@unifi.it (S.S.); benedetta.nacmias@unifi.it (B.N.); 2 IRCCS Fondazione Don Carlo Gnocchi, 50143 Florence, Italy; 3 Regional Referral Centre for Relational Criticalities, 50134 Tuscany Region, Italy; sonia_padiglioni@libero.it; 4 Unit Clinic of Organizations, Careggi University Hospital, 50139 Florence, Italy

**Keywords:** subjective cognitive decline, Alzheimer’s disease, language, visual–spatial ability, executive function, cognitive reserve, *PER2* gene, neuropsychology

## Abstract

Background: Periodic circadian protein homolog 2 (*PER2*) has a role in the intracellular signaling pathways of long-term potentiation and has implications for synaptic plasticity. We aimed to assess the association of PER2 C111G polymorphism with cognitive functions in subjective cognitive decline (SCD). Methods: Forty-five SCD patients were included in this study. All participants underwent extensive neuropsychological investigation, analysis of apolipoprotein E (*APOE*) and *PER2* genotypes, and neuropsychological follow-up every 12 or 24 months for a mean time of 9.87 ± 4.38 years. Results: Nine out of 45 patients (20%) were heterozygous carriers of the *PER2* C111G polymorphism (G carriers), while 36 patients (80%) were not carriers of the G allele (G non-carriers). At baseline, G carriers had a higher language composite score compared to G non-carriers. During follow-up, 15 (34.88%) patients progressed to mild cognitive impairment (MCI). In this group, we found a significant interaction between PER2 G allele and follow-up time, as carriers of G allele showed greater worsening of executive function, visual-spatial ability, and language composite scores compared to G non-carriers. Conclusions: *PER2* C111G polymorphism is associated with better language performance in SCD patients. Nevertheless, as patients progress to MCI, G allele carriers showed a greater worsening in cognitive performance compared to G non-carriers. The effect of PER2 C111G polymorphism depends on the global cognitive status of patients.

## 1. Introduction

Subjective cognitive decline (SCD) is defined as a self-experienced decline in cognitive capacity during which individuals have normal performance on standardized cognitive tests [1]. Recent studies on elderly individuals complaining of cognitive impairment have showed a higher association with neuroradiological features similar to those seen in Alzheimer’s disease (AD) patients, such as volume loss in hippocampal/parahippocampal areas [2] and evidence of amyloid deposition using amyloid positron emission tomography (PET) imaging [3] compared to individuals without SCD. Longitudinal studies have showed that cognitive complaints are linked with subsequent change in hippocampal volume [4], and two meta-analysis suggested that individuals experiencing subjective memory impairment are twice as likely to develop mild cognitive impairment (MCI) or dementia as individuals without [5,6]. Hence, despite SCD being defined on the basis of non-pathological scores at standard neuropsychological assessment, this evidence suggests that SCD should be included into the AD spectrum as an intermediate status between normal cognition and MCI. As a consequence, SCD is getting growing attention as a target population for the identification of Alzheimer’s pathology carriers in a very early stage of the disease. However, the onset of SCD and the risk of progression to MCI and AD is influenced by demographic, cognitive, and genetic factors [7,8,9,10].

Periodic circadian protein homolog 2 (*PER2*), a clock-controlled gene, has a specific role in the intracellular signaling pathways of long-term potentiation (LTP) and has implications for synaptic plasticity and learned behaviors [11]. Therefore, *PER2* is potentially involved in neuropsychological performance and in cognitive changes in the elderly. In a previous study, we focused on the role of *PER2* on cognitive functions in SCD and MCI patients [10]. We found that a polymorphism of *PER2* (C111G, rs2304672) negatively influenced cognitive performance in patients with MCI. In the SCD group, homozygous carriers of the wild-type allele of *PER2* had higher scores on cognitive reserve proxies compared to carriers of the polymorphism. Nevertheless, we detected no association of *PER2* C111G with neuropsychological test scores in SCD patients. In order to explain the lack of association, we hypothesized that, at this stage, the detrimental effect of PER2 C111G polymorphism on cognitive functions might not be effective enough to be detected.

In the present work, we aimed to expand our previous results by testing the effect of *PER2* C111G polymorphism on cognitive function, with respect to composite psychometric measures instead of single neuropsychological scores, and to evaluate the effect of this polymorphism on changes on cognitive performance during the follow-up.

## 2. Materials and Methods

### 2.1. Participants and Clinical Assessment

As part of an ongoing clinical-neuropsychological-genetic survey on SCD, we included 49 consecutive spontaneous patients who self-referred to the Centre for Alzheimer’s Disease and Adult Cognitive Disorders of Careggi Hospital in Florence between October 1998 and May 2014. Inclusion criteria were as follows: (1) complaining of cognitive decline with a duration of ≥6 months; (2) normal functioning on the Activities of Daily Living and the Instrumental Activities of Daily Living scales [12]; (3) diagnosis of SCD according to SCD-I criteria [1]; and (4) unsatisfied criteria for MCI or dementia at baseline [13,14]. Exclusion criteria were as follows: (1) history of head injury, current neurological and/or systemic disease, symptoms of psychosis, major depression, and/or alcoholism or other substance abuse; (2) a follow-up time shorter than two years; (3) a diagnosis of psychiatric or neurological disease that may cause cognitive impairment; and (4) the complete loss of patient data during follow-up.

All participants underwent the following: (1) collection of a comprehensive family and clinical history, general and neurological examination, extensive neuropsychological investigation, and estimation of premorbid intelligence and assessment of depression; (2) peripheral blood collection in order to analyze apolipoprotein E (*APOE*) and *PER2* genotypes; and (3) clinical and neuropsychological follow-up every 12 or 24 months. A positive family history was defined as one or more first-degree relatives with documented cognitive decline.

From the initial sample (49 patients), we excluded two patients who were diagnosed with psychiatric disturbs, one patient with fronto-temporal dementia [15], and one patient with vascular dementia [16]. Ultimately, we included 45 subjects in the analysis.

On the basis of progression from SCD to MCI, patients were classified as “progressed SCD” (pSCD) or “not progressed SCD” (npSCD).

The study was approved by the local institutional review board. All participants gave their written informed consent. All procedures involving experiments on human subjects were done in compliance with the ethical standards of the Committee on Human Experimentation of the institution according to the Helsinki Declaration of 1975 and with applicable domestic laws.

### 2.2. Neuropsychological Assessment

All subjects were evaluated at baseline and every 12 or 24 months by a comprehensive neuropsychological battery as described in more detail elsewhere [17]. Cognitive complaints were explored at baseline using a survey based on the Memory Assessment Clinics-Questionnaire (MAC-Q) [18]. Composite scores for cognitive domains were obtained by arithmetic mean of single test *z*-scores (calculated according to normative data of the Italian population). In more detail, we considered the following composite scores of cognitive domains: long-term verbal memory, a combination of Five Words Recall and Paired Words Recall after 10 min and after 24 h, Babcock Short Story Recall; executive function: Trail Making test (part B-A); language, a combination of the Token Test and Phonemic Fluency test; visuospatial abilities, a combination of the Trail Making test (part A) and copy of Rey–Osterrieth complex figure test; visuospatial long-term memory (recall of Rey–Osterrieth complex figure test); working memory, and a combination of the digit span and *Corsi* Block-Tapping Test.

### 2.3. APOE ε4 and PER2 C111G Genotyping

A standard automated method (QIAcube, QIAGEN) was used to isolate genomic DNA from peripheral blood samples. *APOE* genotypes were investigated by HRMA (high-resolution melting analysis). Two sets of PCR primers were designed to amplify the regions encompassing rs7412 (NC_000019.9:g.45412079C>T) and rs429358 (NC_000019.9:g.45411941T>C). The *APOE* genotype was coded as *APOE* ε4^−^ (no *APOE* ε4 alleles) and *APOE* ε4^+^ (presence of one or two *APOE* ε4 alleles).

The analyses of the *PER2* C111G polymorphism were performed using HRMA, with the following primers: forward 5′-ACAGAAAGAGTCAAATGGGTGC-3′, reverse 5′-TGTCCACATCTTCCTGCAGT-3′ with an annealing temperature of 60 °C. The samples with known *PER2* genotypes, which were validated by DNA sequencing, were used as standard references. Patients who were carriers of the polymorphism were classified as “G carriers”, while patients who were not carriers of the polymorphism were classified as “G non-carriers”.

### 2.4. Statistical Analysis

Scores at cognitive tests were reported as *z*-scores calculated by mean and standard deviation (*SD*) with respect to the Italian general population reported in literature for each neuropsychological test [17,19,20,21,22,23]. We tested for the normality distribution of the data using the Shapiro–Wilk test. Patient groups were characterized by using means and *SD*, median and interquartile range (IQR), and frequencies or percentages and 95% confidence interval (95% CI) for continuous distributed variables, continuous non-normally distributed variables, and categorical variables, respectively. We used *t*-tests or non-parametric Mann–Whitney U tests for between groups’ comparisons, Pearson’s correlation coefficient or non-parametric Spearman’s *ρ* (rho) to evaluate correlations between groups’ numeric measures, and chi-square tests to compare categorical data. We used binomial logistic regression for multivariate analysis. To estimate the effect of *PER2* polymorphism on neuropsychological test scores over time, we used generalized estimating equations. All statistical analyses were performed with SPSS software v.25 (SPSS Inc., Chicago, IL, USA) and R 4.0.3 (R Foundation for Statistical Computing, Vienna, Austria, 2013).

## 3. Results

### 3.1. Description of the Sample and Comparison of Demographic Features between G Carriers and G Non-Carriers

Of the whole sample, 33 patients were women and 12 were men. The mean age at baseline evaluation was 61.26 ± 8.65 years. Thirteen patients (37.14% [95% CI 22.70–51.78]) were carriers of the *APOE* ε4 allele (*APOE* ε4^+^). Nine out of 45 patients (20.00% [95% CI 8.04–31.96]) were carriers of the CG genotype (G carriers), while 36 patients (80.00% [95% CI 68.04–91.96%]) were carriers of the CC genotype (G non-carriers). None of the patients was carrier of the GG genotype. The genotypic distribution of *PER2* gene was in Hardy–Weinberg equilibrium (χ^2^ = 0.56, *p* > 0.05). We found no difference between G carriers and G non-carriers with respect to age at baseline, disease duration (time from onset of symptoms and baseline evaluation), sex, score of scale for depression, *APOE* ε4 allele frequency, Mini-Mental State Examination (MMSE). G carriers had less years of education (8.00 [IQR 7.00] vs. 12.00 [IQR 9.00], U = 75.50, *z* = −12.47, *p* = 0.012) as compared to G non-carriers (Table 1). No differences in single neuropsychological *z*-scores were found between G carriers and G non-carriers (Supplementary Table S1).

### 3.2. Comparison of Neuropsychological Composite Scores between G Carriers and G Non-Carriers at Baseline

**Figure 1:**
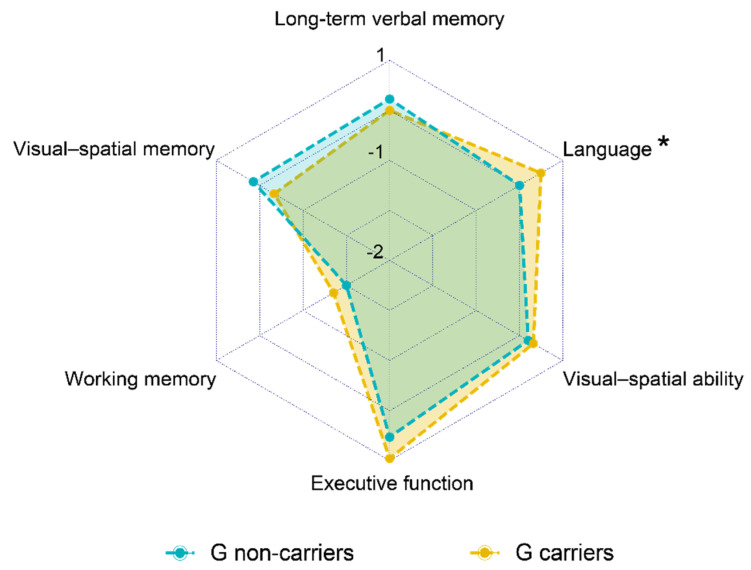
Comparison of composite score for each cognitive domain between G carriers and G non-carriers. Values quoted in the axis are median values. Language composite score (*) was higher in G carriers as compared to G non-carriers (0.25 [IQR 0.94] vs. 0.62 [IQR 0.52], *p* = 0.018).

G carriers had higher language composite score compared to G non-carriers (0.25 [IQR 0.94] vs. 0.62 [IQR 0.52], U = 237.5, *z* = 2.33, *p* = 0.018). No difference with respect to the other cognitive domains were found between G carriers and G non-carriers (Table 1, Figure 1).

### 3.3. Multivariate Analysis

In order to ascertain that the difference in language composite score between G carriers and G non-carriers was independent from possible confounding variables, we carried out a binomial logistic regression. We considered language, years of education, age, sex, disease duration, and *APOE* genotype as covariates and *PER2* C111G genotype as a dependent variable. The regression model was statistically significant (χ^2^ = 18.0, *p* = 0.006). The model explained 52.7% (Nagelkerke R2) of the variance in conversion and correctly classified 88.6% of cases. Of the covariates considered for the analysis, only lower education (Wald = 4.05, *p* = 0.044, OR = 0.71 [95% CI 0.50–0.99]) and higher language score (Wald = 4.92, *p* = 0.027, OR = 25.79 [95% CI 1.44–462.50]) were significantly associated with a higher proportion of G allele (Table 2).

### 3.4. Longitudinal Association of PER2 Genotype with Cognitive Performance

Patients were followed-up for a mean time of 9.87 ± 4.38 years. During this period, 15 (34.88% [95% CI 20.63–49.13]) patients progressed to MCI (pSCD). Thirty patients (65.12% [95% CI 50.87–79.37]) did not progress. Two out of 15 pSCD patients (13.33%) and five out of 30 npSCD patients (16.67%) were G carriers. There were no differences between pSCD and npSCD groups with respect to age at baseline, disease duration, follow-up time, years of education, MMSE, Hamilton Depression rating scale (HDRS), MAC-Q, and *APOE* ε4 and *PER2 G* allele proportion. No differences in cognitive domain composite scores were found between G carriers and G non-carriers at the last follow-up evaluation (Table 3).

In order to explore the effect of *PER2* polymorphism on changes on cognitive performance during follow-up, we ran generalized estimating equations. We considered baseline evaluation and last follow-up neuropsychological evaluation as within-subjects variables, composite *z*-scores of each cognitive domain as dependent variables, and *PER2* genotype (G carrier or G non-carriers) and follow-up time (years between first and last neuropsychological evaluation) as predictors. The model included follow-up time and interaction of follow-up time with *PER2* genotype.

For the whole sample, we did not find any effect of *PER2* polymorphism on changes in cognitive performance during the follow-up (Table 4).

**Figure 2:**
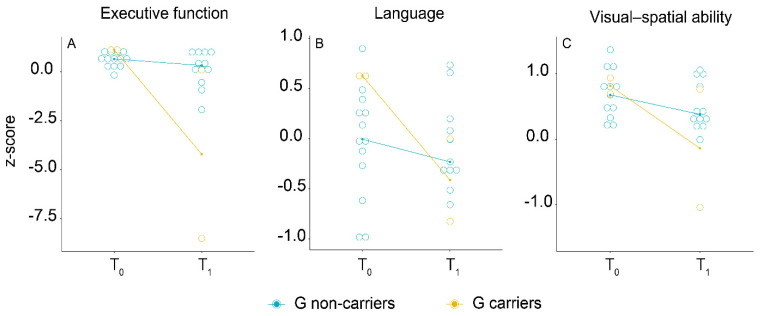
Longitudinal association of PER2 genotype with composite scores for executive function (**A**), language (**B**), and visual–spatial ability (**C**) in the “progressed SCD” (pSCD) group. On the *x*-axis, T0 indicates the baseline evaluation and T1 indicates the last neuropsychological evaluation. On the *y*-axis, median of composite scores (*z*-score) for each cognitive domain are reported.

We ran the same analysis on pSCD and npSCD separately. No significant differences with respect to demographic features, follow-up time, HDRS, MAC-Q, *APOE* ε4, and MMSE were found between G carriers and G non-carriers at baseline. During follow-up, pSCD patients who were carriers of G allele showed greater worsening of executive function (0.921 [95% CI 0.897:0.946], *p* < 0.001, Figure 2A), language (β = 0.560 [95% CI 0.475:0.660], *p* < 0.001, Figure 2B), and visual–spatial ability (β = 0.885 [95% CI 0.827:0.946], *p* < 0.001, Figure 2C) composite scores compared to G non-carriers (Table 5). We did not find any effect of *PER2* polymorphism on changes in cognitive performance during the follow-up in the npSCD subsample.

## 4. Discussion

The potential role of PER2 on cognitive functions has been suggested by a number of studies on in vitro and mouse models [24,25]. However, the role and the mechanism of *PER2* in neurobiological activities is still poorly understood. *PER2* is one of the core genes of the circadian clock and has a role in generating circadian rhythms [26,27]. In the brain, it is mainly expressed in the suprachiasmatic nucleus of the hypothalamus, midbrain, and forebrain [28,29]. Furthermore, it has been linked with the modulation of the release of neurotransmitters such as dopamine [29,30], glutamate [31,32], GABA [33], and serotonin [34,35]. More recently, a number of studies searched for a role of *PER2* in neurodegenerative disease, with conflicting results observed: *PER2* expression was attenuated in AD mouse models, but studies on humans failed to find an association between *PER2* and AD [36,37,38]. Our results provide clues for a role of *PER2* in cognition in humans as well. Nevertheless, its effect may be much more complex than being just a risk or protective factor, with a relationship described by a non-linear function.

As the first result, we showed that *PER2* C111G polymorphism is associated with cognitive functions in SCD patients. We recently showed that *PER2* C111G polymorphism has a detrimental role on single neuropsychological test scores assessing for language, memory, executive function, and visual–spatial ability in patients with MCI [10]. In the present analysis, we found an opposite effect of this polymorphism in patients with SCD, as carriers of the polymorphism performed better than non-carriers on a language composite score. This evidence may appear in contrast with our previous findings. Nevertheless, as a preliminary result on a subgroup of patients, we underlined that among patients who progressed to MCI, G allele carriers showed a greater worsening in language, executive function, and visual-spatial ability.

This result may suggest that the effect of the *PER2* variant on cognitive functions depends on the global cognitive status of subjects. In other words, we might hypothesize that cognitively healthy subjects who are carriers of the *PER2* C111G polymorphism have an advantage with regard to linguistic performance, but this effect might disappear in the progression to MCI. A similar phenomena has been described for a polymorphism of *BDNF* gene [39], and resembles the dimorphic effect of cognitive reserve on progression from SCD to MCI and from MCI to AD [7,40]. Our results precisely showed that a relationship exists between *PER2* and cognitive reserve. Indeed, studies on mouse models found that *PER2* is involved in the mechanism of long-term potentiation, synaptic plasticity, and learned behaviors [11]. Therefore, our results may suggest an implication of the *PER2* gene on a mechanism underling cognitive reserve in humans. Further studies on this topic, especially those including a larger number of patients, will provide crucial findings to expand our knowledge of the neurobiological mechanism of neuropsychological processes and the development of cognitive reserve.

This study has remarkable strengths. First of all, we would like to emphasize that this is the first study assessing the effect of *PER2* C111G polymorphism on cognition in SCD patients. Secondly, in addition to neuropsychological scores, our analysis included other measures estimating features involved in cognitive performance (cognitive reserve, mood state, and cognitive complaint). This provides useful data to exclude possible confounding factors and to support our findings. Finally, our longitudinal analyses are based on data from a very long follow-up time.

The small size of our cohort of patients is the first limitation of our study. In particular, only nine patients were G carriers in the whole sample, and only two patients were G carriers in the pSCD group. This limits our conclusions and means that, in order to ground our findings, we shall expand our sample. Also, we must point out that, despite a long mean follow-up time, we encountered a high variability in follow-up times, as shown by the *SD* value of this variable. Another limitation is the lack of AD biomarkers data. Finally, as it is a single-center study, there may be biases with regard to assessment and diagnosis procedures.

## 5. Conclusions

*PER2* C111G polymorphism differently influences cognitive functions according to the global cognitive status of the subject. In particular, the *PER2* G allele seems to provide an advantage in relation to language in cognitively healthy subjects. Nevertheless, in patients who progress to objective cognitive impairment, this advantage seems to disappear, and the G allele is associated with a greater decline in language, executive function, and visual–spatial ability. Our findings pave the way to future research on the implication of the *PER2* gene on the neurobiological mechanism(s) underling cognitive reserve, as well as the neuropsychological functions in healthy and in cognitively impaired patients and in neurodegenerative diseases.

## Figures and Tables

**Table 1:** Comparison of demographic, cognitive, and genetic features between G carriers and G non-carriers.

Variable	G Non-Carriers	G Carriers	*p*
*N*	36	9	
Age at baseline, median (IQR)	60.80 (17.24)	62.44 (9.81)	0.758
Disease duration, median (IQR)	3.82 (2.92)	3.33 (4.24)	0.092
Sex (no. women/no. men)	25/11	8/1	0.241
Education, median (IQR)	12.00 (9.00)	8.00 (7.00)	0.016
*APOE* ε4^+^, % (95% CI)	30.55 (16.78–44.32)	22.22 (9.79–34.65)	0.625
MMSE, median (IQR)	27.00 (3.70)	26.61 (4.70)	0.251
HDRS, median (IQR)	4.00 (6.00)	5.50 (4.00)	0.303
MAC-Q, median (IQR)	25.00 (3.00)	25.50 (4.00)	0.326
Long-term verbal memory, median (IQR)	0.42 (0.54)	0.25 (0.62)	0.244
Working memory, median (IQR)	−1.25 (1.61)	−1.03 (1.61)	0.770
Visual–spatial memory, median (IQR)	0.36 (0.83)	0.00 (0.18)	0.163
Visual–spatial ability, median (IQR)	0.40 (±0.61)	0.49 (±0.47)	0.694
Executive function, median (IQR)	0.65 (±0.40)	0.97 (±0.72)	0.305
Language, median (IQR)	0.25 (0.94)	0.62 (0.52)	0.018

Values quoted in the table are mean and standard deviation (*SD*), medians and interquartile ranges (IQRs), frequencies or percentages, and 95% CI. Age at baseline, disease duration, and education are expressed in years. Cognitive composite scores are expressed as *z*-scores. *p* indicates level of significance for comparison between G carriers and G non-carriers (statistical significance at the *p* < 0.05). Abbreviations: MMSE: Mini-Mental State Examination; HDRS: Hamilton Depression rating scale; MAC-Q: Memory Assessment Clinics-Questionnaire.

**Table 2:** Logistic regression model for multivariate analysis.

	B	Wald	*p*	OR	95% C.I.	
					Lower	Upper
	3.32	4.92	0.027	25.79	1.44	462.50
Education (years)	−0.345	4.05	0.044	0.71	0.50	0.99
Age (years)	−0.05	0.48	0.49	0.95	0.82	1.10
Disease duration (years)	−0.30	2.16	0.14	0.74	0.49	1.11
Female sex	0.35	0.06	0.80	1.41	0.10	20.22
*APOE* ε4^+^	0.43	0.13	0.72	1.53	0.15	16.06
Constant	4.82	0.81	0.40	124.46		

Regression coefficients (B), Wald coefficients, *p*-values (*p*), odds ratios (ORs), and 95% confidence intervals (95% CIs) are shown. All the covariates included in the analysis are reported. *PER2* C111G genotype (G or CC) was considered a dependent variable. The regression model was statistically significant (χ^2^ = 18.0, *p* = 0.006). Language and education were statistically significant (significant differences at *p* < 0.05).

**Table 3:** Comparison of the demographic, cognitive, and genetic features between G carriers and G non-carriers at the last follow-up evaluation.

Variable	G Non-Carriers	G Carriers	*p*
*N*	36	9	
Long-term verbal memory, median (IQR)	0.07 (0.94)	0.13 (0.87)	0.547
Working memory, median (IQR)	−1.25 (1.03)	−1.25 (0.79)	0.833
Visual-spatial memory, median (IQR)	0.82 (1.19)	0.22 (2.08)	0.276
Visual-spatial ability, median (IQR)	0.50 (0.71)	0.76 (0.99)	0.323
Executive function, median (IQR)	0.51 (0.71)	0.37 (1.91)	0.838
Language, median (IQR)	0.20 (0.86)	0.18 (0.46)	0.850

Values quoted in the table are mean and standard deviation (*SD*), medians and interquartile ranges (IQRs), frequencies or percentages, and 95% CI. Age at baseline, disease duration, and education are expressed in years. Cognitive composite scores are expressed as *z*-scores. *p* indicates level of significance for comparison between G carriers and G non-carriers (statistical significance at the *p* < 0.05).

**Table 4:** Generalized estimating equations as a function of *PER2* genotype over follow-up time.

	Follow-Up Time	Follow-Up Time × G Allele
	β (95% CI)	β (95% CI)
Long-term verbal memory	0.942 (0.889:0.999)	1.016 (0.918:1.125)
Working memory	1.013 (0.973:1.054)	0.971 (0.922:1.023)
Visual–spatial memory	1.034 (0.986:1.085)	0.987 (0.856:1.138)
Visual–spatial ability	1.030 (0.993:1.068)	0.973 (0.874:1.083)
Executive function	0.974 (0.944:1.006)	0.335 (0.595:1.193)
Language	0.995 (0.975:1.015)	0.971 (0.923:1.021)

β coefficients and 95% confidence intervals (95% CIs) for model including time and time × G allele are reported. There was no significant effect of *PER2* polymorphism on changes in cognitive performance during the follow-up.

**Table 5:** Generalized estimating equations for neuropsychological scores as a function of *PER2* genotype over follow-up time.

Cognitive Domains	Time	Time × G Allele
	β (95% C.I)	β (95% C.I)
Long-term verbal memory	0.823 (0.785:0.862) ***	1.049 (0.995:1.106)
Working memory	1.017 (0.972:1.063)	0.449 (0.108:1.866)
Visual–spatial memory	0.987 (0.938:1.038)	0.980 (0.788:1.006)
Visual–spatial ability	0.977 (0.954:1.047)	0.885 (0.827:0.946) ***
Executive function	0.979 (0.954:1.005)	0.921 (0.897:0.946) ***
Language	0.941 (0.885:1.001)	0.560 (0.475:0.660) ***

β coefficients and 95% confidence intervals (95% CIs) for model including time and time × G allele are reported. *** *p* < 0.001.

## Data Availability

Anonymized data that support the findings of this study will be shared by request from any qualified investigator.

## Supplementary Materials

The following are available online at https://www.mdpi.com/article/10.3390/diagnostics11040718/s1.
