# Supplementary material for: Dual Effect of PER2 C111G Polymorphism on Cognitive Functions across Progression from Subjective Cognitive Decline to Mild Cognitive Impairment
Source: Diagnostics (Basel). 2021 Apr 18;11(4):718. doi: 10.3390/diagnostics11040718 (PMC8074126; doi:10.3390/diagnostics11040718)
Supplement: Supplementary file 1 [file diagnostics-11-00718-s001.zip › diagnostics-1176891-supplementary.pdf]

Table S1. Comparison neuropsychological z-scores between G carriers and G non-carriers

Tests	G non-carriers	G carriers	p
N	36	9	
Five Words Acquisition	0.10 (1.45)	0.10 (1.58)	0.748
Five Words Recall after 10 minutes	0.21 (1.58)	0.64 (1.58)	0.856
Five Words Recall after 24 hours	-0.08 (0.95)	-0.08 (0.50)	0.625
Paired Words Acquisition	0.68 (2.00)	-0.12 (1.20)	0.104
Paired Words Recall after 10 minutes	1.00 (0.71)	1.00 (1.43)	0.586
Paired Words Recall after 24 hours	0.23 (1.61)	-0.09 (1.61)	0.944
Babcock short story	0.21 (0.89)	-0.26 (1.55)	0.232
Babcock short story recall	0.96 (0.95)	-0.44 (2.10)	0.306
Digit Span	-0.54 (1.83)	-0.77 (2.29)	0.944
Corsi Tapping Test	-1.27 (2.29)	-1.27 (1.83)	0.548
Trail Making test part A	0.41 (0.85)	0.77 (1.28)	0.148
Trail Making test part B	0.55 (0.54)	1.09 (0.61)	0.148
Trail Making test B-A	0.62 (0.54)	0.97 (0.72)	0.252
Token test	0.41 (0.62)	0.72 (0.78)	0.131
Phonemic Fluency Test	-0.15 (1.23)	0.43 (0.70)	0.081
Rey-Osterrieth complex figure - copy	0.81 (0.56)	0.37 (0.55)	0.472
Rey-Osterrieth complex figure - recall	0.46 (1.04)	0.01 (1.35)	0.224

Values quoted in the table are medians and interquartile ranges (IQR). *p* indicates level of significance for comparison between G carriers and G non-carriers (statistical significance at the $p < 0.05$).
